# Harvest of the Oleaginous Microalgae *Scenedesmus obtusiusculus* by Flocculation From Culture Based on Natural Water Sources

**DOI:** 10.3389/fbioe.2018.00200

**Published:** 2018-12-18

**Authors:** Felix Bracharz, Daniel Helmdach, Isabel Aschenbrenner, Nils Funck, Daniel Wibberg, Anika Winkler, Frank Bohnen, Jörn Kalinowski, Norbert Mehlmer, Thomas B. Brück

**Affiliations:** ^1^Werner Siemens Chair of Synthetic Biotechnology, Department of Chemistry, Technical University Munich (TUM), Munich, Germany; ^2^Center for Biotechnology-CeBiTec, University Bielefeld, Bielefeld, Germany; ^3^BBSI GmbH, Gfällach, Germany

**Keywords:** microalgae, harvest, flocculation, *Scenedesmus obtusiusculus*, tannin, chitosan

## Abstract

Harvest and dewatering poses a significant economical burden for industrial algae biomass production. To mitigate these effects, energy efficient techniques for these process steps have to be developed. Flocculation of the microalgae *Scenedesmus obtusiusculus* in salt based medium was induced by pH-shift and alternatively by addition of two biological flocculants, chitosan, and the commercial tannin CFL-PT. This is the first time that CFL-PT is used as an algae flocculant particularly focusing on harvesting of halophilic strains. The method was characterized and subsequently optimized. In comparison to biological flocculants, induction by pH shift is far cheaper, but due to buffering effects of the brackish cultivation medium infeasible amounts of base are required to raise the pH-value. tannin appears to be superior compared to chitosan not only in the absence of algae organic matter (AOM), but tannin-based harvest is also more robust regarding culture pH in presence of AOM. A higher flocculant-demand for modified tannin compared to chitosan is offset by the lower price. Given the employed strain and cultivation conditions, cultivation time had no pronounced effect on flocculation efficiencies (FE) while algae zeta-potential and bacterial communities also remained stable.

## Introduction

Due to multiple value-adding compounds including lipids, proteins, and pigments, microalgal biomass is a promising, sustainable feedstock with application in the food, biofuel, and cosmetic industries (Sanghvi and Lo, 2010). Algae production does not compete with food production and is distinct from terrestrial plant biomass due to high areal productivity and space time yields, resulting in a 10- to 50-fold increase in growth rates (Chen et al., 2011; Schlagermann et al., 2012). The study focused on the microalgae *Scenedesmus obtusiusculus* A189, a newly isolated strain of Chlorophyta with high growth rates in fresh and salt water as well as high lipid and carbohydrate content. The maximum CO_2_ fixation rate of *S. obtusiusculus* is among the highest for Scenedesmaceae (Toledo-Cervantes et al., 2013). Thus, this microalga seems to be of great biotechnological interest due to its efficient CO_2_ uptake coupled to high lipid production as well as fast biomass productivity. These aspects may support the potential application as renewable biofuel source (Chisti, 2007).

While biomass production in both open and closed bioreactors systems is becoming increasingly efficient, harvest, and downstream processing of algae biomass remain major challenges in industrial process development. At present, harvesting—the procedure to effectively separate microalgal biomass from culture medium—accounts for up to 30% of the total production cost (Barros et al., 2015). Depending on the target microorganism and the characteristics of the desired product, a single-step concentration or a two-step separation, consisting of thickening, and subsequent dewatering procedures, can be applied (Molina Grima et al., 2003; Brennan and Owende, 2010). Algae biomass harvesting issues mainly result from small cell size, colloidal stability, and low biomass concentrations (Danquah et al., 2009; Uduman et al., 2010).

Thus, a wide array of solutions have been proposed to address these difficulties (Gerardo et al., 2015) including centrifugation (Dassey and Theegala, 2013; Pandey et al., 2013), filtration (Rossignol et al., 1999; Huang et al., 2012), flotation (Hanotu et al., 2012; Coward et al., 2015), and different means of flocculation by employing physical (Cerff et al., 2012; Prochazkova et al., 2013), chemical (Sirin et al., 2012; Chen et al., 2013) or biological means (Salim et al., 2011; Zhou et al., 2013). In view of techno-economic feasibility, none of the current methods combines universal applicability, process robustness, cost-, energy-, and resource efficiency. At present, centrifugation remains the most robust method, but suffers from high energy and labor costs. By contrast, filtration conventionally requires less energy and is more suitable for large cells. However, biofouling remains a potentially costly challenge for this process (Rickman et al., 2012). Beyond that, flocculation-sedimentation, which can also be used for water purification in wastewater treatment, drinking water production, and brewing (Vandamme et al., 2013), is generally regarded as an energy and resource efficient method for harvest of algal biomass (Schlesinger et al., 2012; Pérez et al., 2016), thus representing a suitable option for processing large volumes of culture medium at minimal cost to make commercial application of low-value products feasible (Pienkos and Darzins, 2009).

Flocculation is a process by which particles can be removed from a suspension. Suspensions are known to be stabilized either by steric properties and/or by a surface charge of their suspended particles. For the latter, flocculation is a promising strategy due to initiating particle formation by altering the ionic strength of the medium and thus the charge state of the algae cells. Flocculation is a process by which particles can be removed from a suspension. In the case of algae, flocculation can be induced by overcoming the negative algal surface charge (Pandey et al., 2013). Thereby, the entire surface charge can be neutralized, especially by changing the pH-value (Spilling et al., 2011). Thus, autoflocculation can be achieved by addition of the bases Ca(OH)_2_, NaOH, or KOH, respectively. Furthermore, lowering the surface charge by raising the pH can lead to the attraction of cationic counter ions (specifically Mg^2+^), which attach to the cell surface. Subsequently, other cells attach to this now positively charged surface (Vandamme et al., 2012b). A variation of this is the electrostatic patch mechanism, in which only certain areas on the cell surface are neutralized or modified. This in turn leads to agglomeration of cells at patches with complementary charges. Subsequently, flocs can be formed by bridging of multiple cells, which in turn start to “collect” further algae (Vandamme et al., 2013).

For chemical flocculation, iron or aluminum salts are very common, cheaply available flocculation agents. However, a general issue for flocculation remains in downstream processing: High loadings are typically required for induction of flocculation and flocculants might subsequently pose problems for extractions (synthetic polymers) or restricting the use of the biomass (alum content) (Vandamme et al., 2013). Induction of autoflocculation by shifting pH requires constant pH readjustment and media recycling is problematic with high concentrations of metal ions (Molina Grima et al., 2003). This study focuses on the use of biodegradable, non-toxic polymeric flocculants for efficient algae harvest.

Biological flocculants are cells, cell wall components or biologically derived products, which can be used for flocculation. Commonly, the mechanism of flocculation is the same as for synthetic polymers as bio-derived flocculants are also cationic polymers, but lower dosages are necessary (Chen and Pan, 2012). Furthermore, they are non-toxic, highly biodegradable, and hence in principal suitable for harvest and media recycling of microalgae, which may significantly reduce overall cost of microalgae cultivation (Milledge and Heaven, 2013).

Examples for these biological flocculants include chitosan, xanthan or tannin. Chitosan is obtained by chemical deacetylation of chitin, a natural biopolymer derived from arthropod shells constituting *N*-acetylglucosamine, and glucosamine building blocks. It has been applied as flocculant for over 40 years (Gidas et al., 1970). However, the comparatively high price has prevented its application at technical scales. As a cationic polymer, chitosan can bind algae, and form flocs, which subsequently sediment. In some cases, this process was described to be very efficient (Ahmad et al., 2011) (99% algae removal at 1 mg/L chitosan). Nevertheless, efficiency strongly depends on the specific algae strain and the salt concentration in the medium (Morales et al., 1985) and pH-value (Divakaran and Sivasankara Pillai, 2002). Xanthan, a bacterial exopolysaccaride, has been used as flocculant in fresh water to remove harmful algae blooms (Chen and Pan, 2012). In contrast, few reports have applied the plant derived polyphenol compound tannin as a flocculant, although it is commercially available as de-emulsifier (Separ-Chemie n.d.). To that end, tannin was used in wastewater treatment (Beltrán-Heredia and Sánchez-Martín, 2009) and flocculation of the cyanobacterium *Microcystis aeruginosa* (flocculation efficiencies (FE) >90% w/v), where it was shown to be effective below pH-values of 7 and 9, respectively, depending on the preparation method (Wang et al., 2013). Similarly, the commercial tannin preparation polysepar CFL 25 has previously been applied as a flocculant for the freshwater microalgae *Scenedesmus acuminatus* resulting in 70% w/v FE at a dosing of 70 mg/L (Bleeke et al., 2015). Another tannin-based product, Tanfloc, was previously applied at a concentration of 5 mg/L to harvest the freshwater algae *Chlorella vulgaris* at 250 mg/L dry biomass concentration (Roselet et al., 2012).

Recently, Schulze et al. reported on the isolation of *S. obtusiusculus* and, particularly important, on the optimization of media conditions for carbohydrate and lipid production by reduction of protein content (Schulze et al., 2016). The optimized cultivation medium containing 5% artificial sea salt resulted in a 2-fold increase in the intracellular lipid and carbohydrate content. As the optimized medium composition contains significant amounts of artificial sea salt, harvest by flocculation is challenging due to increased ionic strength. High salt concentration in algae media typically leads to a reduction of the algae cells' surface charge (i.e., zeta potential) (Vandamme et al., 2013). As salts act as buffers, a pH-shift is more difficult to achieve.

As algae harvesting significantly adds to the total production cost of algal biomass (Barros et al., 2015), development of effective and robust flocculation methods is key to design economically viable industrial processes (Riaño et al., 2012; Shen et al., 2013; Surendhiran and Vijay, 2014). Nonetheless, flocculation process optimization is complex and time-consuming as it strongly depends on the selected algae strain, media composition, culture age, and cell density as well as the specific flocculant (Vandamme et al., 2012a). Studies on flocculation-based algae harvest mostly focus on standard algae media but not on optimized media. Thus, in this paper we employ autoflocculation and biological flocculants to harvest *S. obtusiusculus* cells grown under optimized conditions in cultivation media containing high salt concentrations. We further attempt to establish the tannin-based biopolymer CFL-PT, which has previously gathered little attention, as a low-cost alternative to chitosan.

In this work, reaction time and pH effects on bioflocculants are evaluated while considering effects of microbial communities on flocculation behavior. Thereby, challenges and interdependencies of factors affecting the process of flocculation could be determined, which have to be considered when working with flocculation-based algae harvest.

## Materials and Methods

### Microalgae Cultivation

*S. obtusiusculus* A189 was obtained from the Pharmaceutical Biology Group of the Ernst-Moritz-Arndt University in Greifswald (Schulze et al., 2016). For cultivation, an optimized medium (ABV medium), containing in g/L: NaNO_3_ 0.375, K_2_HPO_4_ 0.4, C_6_H_9_FeNO_7_ 0.0006, Artificial Salt Water 5, as well as a modified BG11 (herein referred to as BG11S), containing NaNO_3_ 1.5, K_2_HPO_4_ 0.04, MgSO_4_ · 7 H_2_O 0.075, CaCl_2_ · 2 H_2_O 0.036, C_6_H_8_O_7_ 0.006, Na-EDTA 0.001, C_6_H_8_O_7_ · C_6_H_9_FeNO_7_ 0.006, Na_2_CO_3_ 0.02, and 1 mL/L of trace element solution, were employed. Trace elements contained in g/L: H_3_BO_3_ 6.1, MnSO_4_ 22.3, ZnSO_4_ · H_2_O 28.7, CuSO_4_ · 5 H_2_O 0.25, (NH_4_)_6_Mo_7_O_24_ · 4 H_2_O 1.25. Artificial salt water was obtained from Tropic Meeresaquaristik. The composition of the artificial salt water base salt is shown in Supplemental 1.

For evaluating harvest by pH-shift, tannin and chitosan in fresh- and brackish water, 3 L of the respective medium were sterile filtered, added to the reactor and cultures were inoculated to an OD_680_ of 0.1–0.2. Regulation of pH to 8 was done by CO_2_ injections into the culture. Air stream was set at 1 L/L culture/h and stirrer was at 400 rpm. Irradiation was set at 10%, ~74 μMol photons m^−2^s^−1.^

For the evaluation of growth phase dependency and effects of algae organic matter (AOM), algae were cultivated in an aerated 30 L bubble column reactor under a constant stream of air at ~275 L/min. A Milwaukee MC122 pH sensor was used to stabilize the pH at 8 by controlling CO_2_ injections into the air stream. Culture growth and condition were monitored using OD_680_ and microscopy. Temperature was at 28 ± 2°C and cultures were continuously irradiated with fluorescent tubes at ~240 μMol photons m^−2^s^−1^.

### Biomass Determination

Dry biomass concentration (DBC) was determined through a correlation of biomass to OD_680_ measured with a Genesys 103 UV-VIS (Equation 1, Supplemental 2). If not stated differently, values were measured as triplicates.

$$\begin{array}{l} \text{DBC}=0.485^{*}{\text{OD}}_{680}+0.0879\\ {\text{R}}^{2}=0.998 \end{array}$$

For the calibration curve, biomass concentration was determined by centrifuging 10 ml culture at 8,000 rpm for 10 min, washing with ddH_2_O and drying to constant weight in an Ohaus M325 Infrared Scale.

### Flocculation Procedure

The flocculation procedure was carried out according to previously published sources with a focus on comparing the flocculants (Castrillo et al., 2013). The process of flocculation including rpm and number of stirring steps was kept constant and was not further optimized. Flocculation was evaluated using a VELP Scientifica JLT4 jar test apparatus. For each test, 150 mL of algae culture were pH adjusted in a beaker and the respective flocculant was added. Algae suspensions were stirred at 300 rpm for 3 min and then left for settling. Flocculation efficiency (FE, η) was determined after 30 min by sampling and determining OD_680_ 2 cm below suspension surface.

$$\begin{array}{l} \;\eta =1-(OD_{\text{f}}/OD_{\text{i}})\\ \text{with }{\text{OD}}_{\text{f}}\text{ being }{\text{OD}}_{680}\text{ after flocculation and }{\text{OD}}_{\text{i}}\text{ as}\\ \text{ initial }{\text{OD}}_{680} \end{array}$$

Chitosan was obtained from BioLog Heppe GmbH (Biolog Heppe GmbH, 2017) and was prepared as 10 g/L stock solution in 1% v/v acetic acid. Modified Larch-Tannin (Polysepar CFL-PT), supplied as quaternary, cationic ammonium-tannate, was obtained from Separ Chemie (Separ-Chemie, 2017). CFL-PT is marketed as emulsion breaker and fixation agent for the treatment of wastewater or process water. According to REACH specification noted in the respective data sheet (Separ-Chemie, 2015), it exhibits low toxicity (LD50 oral: 2260 mg/kg for *Rattus rattus* and LC50 73.9 mg/L for *Labeo rohita*) and is “easily biodegradable.” For removal of AOM, samples were centrifuged at 6,000 × g for 15 min and resuspended in fresh medium (Henderson et al., 2010).

### Statistical Analysis

R was used for statistical analysis (R Core Team, 2014) including the software packages grofit (Kahm et al., 2010), MASS (Venables and Ripley, 2002), ggplot2 (Wickham, 2009), readr (Wickham et al., 2017), and cowplot (Wilke, 2016), respectively. Parameter selection of multivariate models was carried out according to the Akaike information criterion. Pareto-plots were based on the package pid (Dunn, 2015). Datasets were checked for normality and models for heteroscedasticity.

To evaluate the effect of different bases on autoflocculation (section Harvesting Using pH-Shift), multiple linear regression was applied using settling time (15, 30, 60, 120 min) and pH-value (10, 11, 11.2, 11.4, 11.6, 11.8, 12) as independent variables for each of the bases in a range between pH 11 and 12. Comparison of chitosan and tannin in different media (section Biological Flocculants: Tannin and Chitosan in Fresh- and Brackish Water) was evaluated with Welch's *t*-test. To model chitosan and tannin FE (Section Growth Phase Dependency of Biological Flocculants) at different stages of the growth phase (at 20, 145, 170, 195, 220 h), with different pH-values (8, 9) and varying flocculant concentrations (40, 80 mg/L) a full factorial design was applied. In the last set of experiments, at *t* = 220 h, the presence of AOM was additionally controlled (section Effect of Algae Organic Matter) and tested with Welch's *t*-test.

### High-Throughput 16S rRNA Gene Amplicon Processing and Sequencing

To get first insights into the algae community compositions, high-throughput sequencing of 16S rRNA gene amplicons was performed as described elsewhere (Maus et al., 2017). The primers Pro341F (5′-CCTACGGGGNBGCASCAG-3′) and Pro805R (5′-GACTACNVGGGTATCTAATCC-3′) were used to amplify the hypervariable 16S rRNA regions V3 and V4 of diverse bacteria and archaea (Takahashi et al., 2014). In addition, the primer pair also covers the 16S rRNA gene of some algal chloroplasts and other eukaryotic mitochondrial genomes. In the next step, multiplex identifier tags and Illumina-specific sequencing adaptors were applied for a two-step PCR approach. Only PCR products featuring a size of around 460 bp were purified using AMPureXP® magnetic beads (Beckman Coulter GmbH, Brea, California, USA). Obtained 16S rRNA gene amplicons were qualitative and quantitative analyzed by using the Agilent 2100 Bioanalyzer system (Agilent Inc., Santa Clara, California, USA). For paired-end sequencing on the Illumina MiSeq system (Illumina, San Diego, California USA), the generated amplicons were pooled in equimolar amounts. The amplicon raw data was deposited in the EMBL database (Bioproject ID PRJEB22943).

### Quality Control and Amplicon Processing of 16S rRNA Genes

An in-house pipeline, as described recently (Wibberg et al., 2016), performed adapter, and primer trimming for all samples. For amplicon processing, another pipeline including FLASH (Magoc and Salzberg, 2011), USEARCH 8.1 (Edgar, 2010), UPARSE (Edgar, 2013), and the RDP classifier (Wang et al., 2007) was used as described recently (Theuerl et al., 2015; Liebe et al., 2016; Maus et al., 2017). In brief, all sequences that were not merged by FLASH (default settings with one modification: −M 300) were filtered out. In addition, sequences containing ambiguous bases (Ns) and with expected errors >0.5 were also discarded. Afterwards, resulting data was processed and operational taxonomic units were clustered based on the program USEARCH 8.1. These units were taxonomically classified using the RDP classifier (Version 2.9) in 16 S modus. Only hits featuring a confidence value >0.8 were considered. Finally, obtained raw sequence reads were mapped back onto the operational taxonomic unit sequences in order to get quantitative assignments.

## Results and Discussion

### Harvesting Using pH-Shift

Autoflocculation by alkaline pH shift has previously shown to be a cheap and robust method for flocculation (Castrillo et al., 2013). Supplemental 3 shows raw data values of FE for autoflocculation experiments induced by alkaline conditions with different mineral bases at various pH-values. While the FE increases under alkaline pH conditions, magnesium hydroxide could not be adequately dissolved to significantly induce flocculation. Notably, the increasing FE correlates linearly with sedimentation time, if flocculation was induced. For better characterization of the flocculation behavior, individual data sets of effective flocculations (FE > 0.6) were subjected multiple linear regression analysis (Supplemental 4).

**Figure 1:**
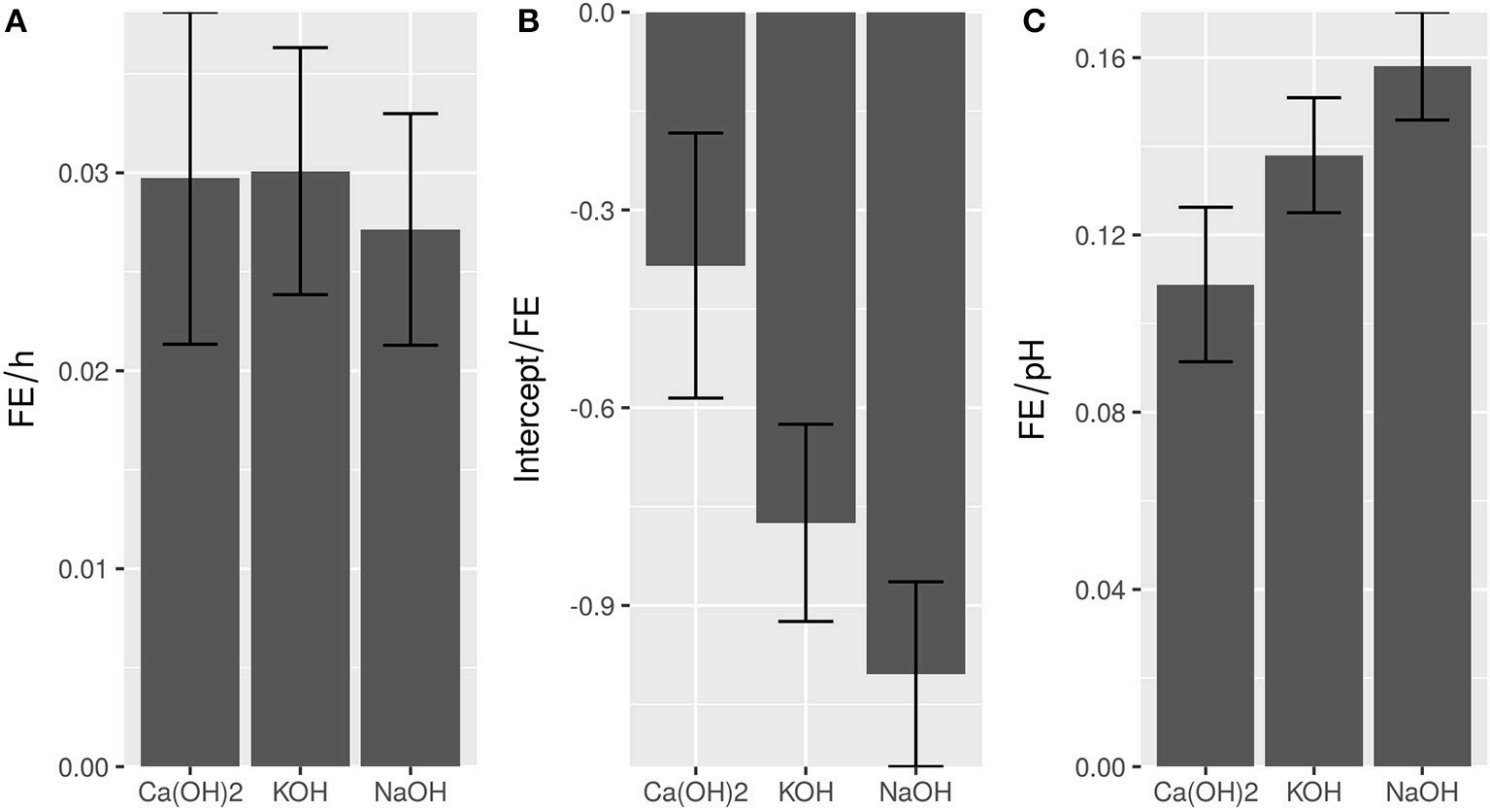
Comparison of regression parameters time **(A)**, intercept **(B)**, and pH **(C)** of different bases. Error bars show standard error of the respective parameter. *R*^2^-values are 0.88 (CaOH_2_), 0.95 (KOH), and 0.96 (NaOH), respectively.

A simple, linear model following FE = pH + time adequately described flocculation behavior for the minerals Ca(OH)_2_, KOH, and NaOH with *R*^2^ = 0.88, 0.95, and 0.95, respectively (Supplemental 4). The impact of the sedimentation time in FE per hour was not affected by the type of flocculant (Figure 1A). This indicated that equivalent flocculation mechanisms, independently of the choice of base, applied. In contrast, Ca(OH)_2_ had a higher intercept than the other flocculants (Figure 1B). This suggests, that the respective flocculant leads to higher FE assuming the same pH and sedimentation time, which is supported by the raw values (Supplemental 3). The higher FE of Ca(OH)_2_ is especially visible at lower pH (Supplemental 3), however with increasing pH, the difference between flocculants disappears, due to the higher effect of pH when using KOH or NaOH (Figure 1C). Correspondingly, at the highest tested pH-value 12 no significant differences (α = 0.05) were observed between the different flocculation agents. In summary, the lowest cost was obtained by using slaked lime to induce autoflocculation (Table 1).

**Table 1:** Comparison of ion properties, required base concentration and base loading for pH 10.8 and corresponding cost per kg base in technical quality as well as harvesting cost per ton biomass.

	**Ion properties**		**[Base]**	**[Biomass]**	**Base load**	**Cost**	**Cost**
	**Angstrom**	**pK_**B**_**	**g/L**	**g/L**	**mg/g biomass**	**$/kg agent**	**$/t biomass**
NaOH	1.02	−0.93	0.204	1	204	0.31	63.24
KOH	1.38	−0.7	0.324	1	324	0.65	210.6
Ca(OH)_2_	1	1.37, 2.43	0.385	1	385	0.1	38.5

The obtained results (Table 1) show much higher demand for base than previously published reports (Vandamme et al., 2012a) with *C. vulgaris*, which can be most likely attributed to buffering effects of the salt containing medium. For comparison, using fresh water medium, Vandamme et al. employed 9, 12, and 18 mg base per gram biomass respectively for the three bases NaOH, KOH, and Ca(OH)_2_.

### Biological Flocculants: Tannin and Chitosan in Fresh- and Brackish Water

**Figure 2:**
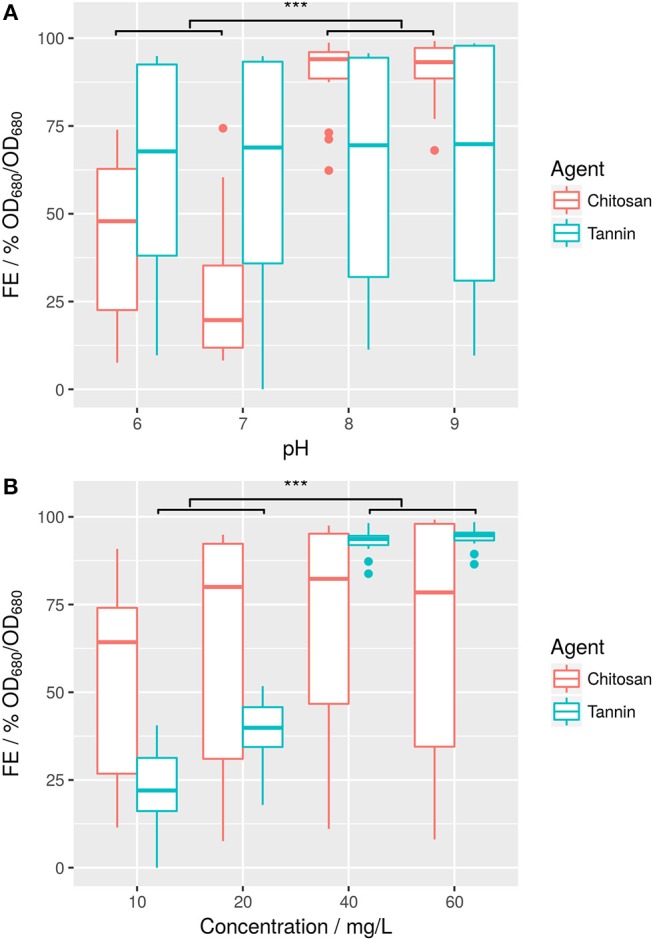
Flocculation efficiencies of flocculation agents chitosan and tannin in ABV medium at different pH-values **(A)** and at different concentrations **(B)**. Welch's *t*-test shows that difference in pH is significant for chitosan (***p ≤ 0.001, 55% FE) and flocculant concentration is significant for tannin (***p ≤ 0.001, difference in means 63% FE). Vice versa, effects are not significant for the other respective flocculant.

Chitosan was first tested in both BG11 and ABV medium. In fresh water medium (BG11), chitosan shows high FE starting at a concentration of 10 mg/L (Supplemental 5). This confirms results of previous reports achieving also high FE of *Scenedesmus* sp. strains with this polymer (Nigam and Ramanathan, 1980; Divakaran and Sivasankara Pillai, 2002; Fierro et al., 2008; Chen et al., 2013). FE plateaus between 0.96 and 0.98 and does not increase even at higher doses up to 40 mg/L. In ABV medium however, flocculation is less stable and apparently pH dependent (Figure 2A). While still active at high pH-values over 8, FE strongly decreases at pH-values between 6 and 8 (Supplemental 6). In comparison to this, tannin-induced flocculation in ABV medium is mostly independent from culture pH even within high salt environment, but in turn depends more on the employed concentration (Figure 2B).

In summary, efficiency of chitosan as flocculant was higher than for tannin in salt water medium: Over 90% FE could be achieved using 20 mg/L chitosan or 40 mg/L tannin. However, cost of the respective tannin ($5/kg) is significantly lower than for chitosan ($25/kg). This results in an overall reduction in cost from $0.5/kg algae biomass to $0.2/kg algae biomass assuming a cell density of approximately 1 g/L.

### Growth Phase Dependency of Biological Flocculants

**Figure 3:**
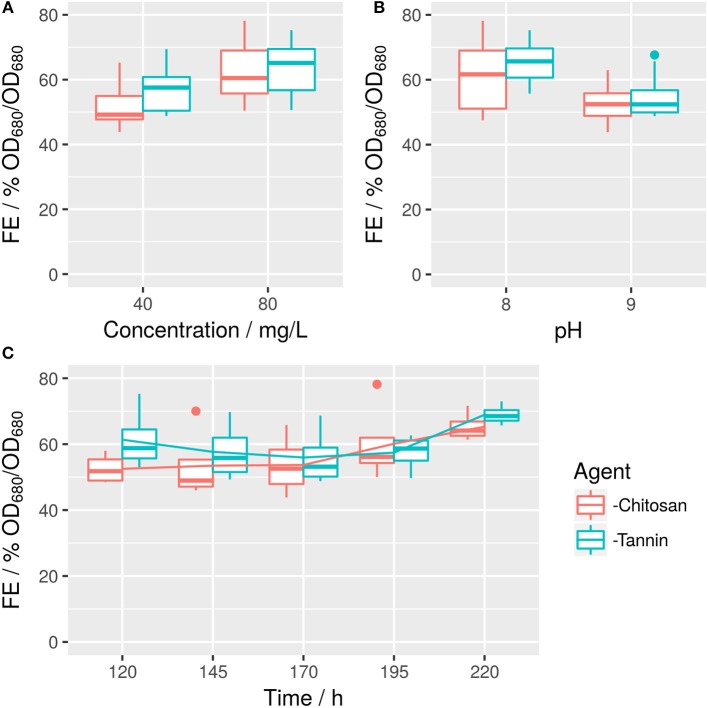
Raw values of tannin- and chitosan-based FE in dependence on concentration **(A)**, pH **(B)**, and time **(C)**. Corresponding effects are described in Figure 4 and Supplemental 8.

*S. obtusiusculus* A189 was cultivated in ABV medium in an open bubble column reactor (Supplemental 7). At five time points, a full factorial design with all combinations of flocculant, flocculant concentration, and pH was conducted (Figure 3). The resulting multivariate model exhibited an *R*^2^ of 0.89 (Supplemental 8 with detailed regression results).

**Figure 4:**
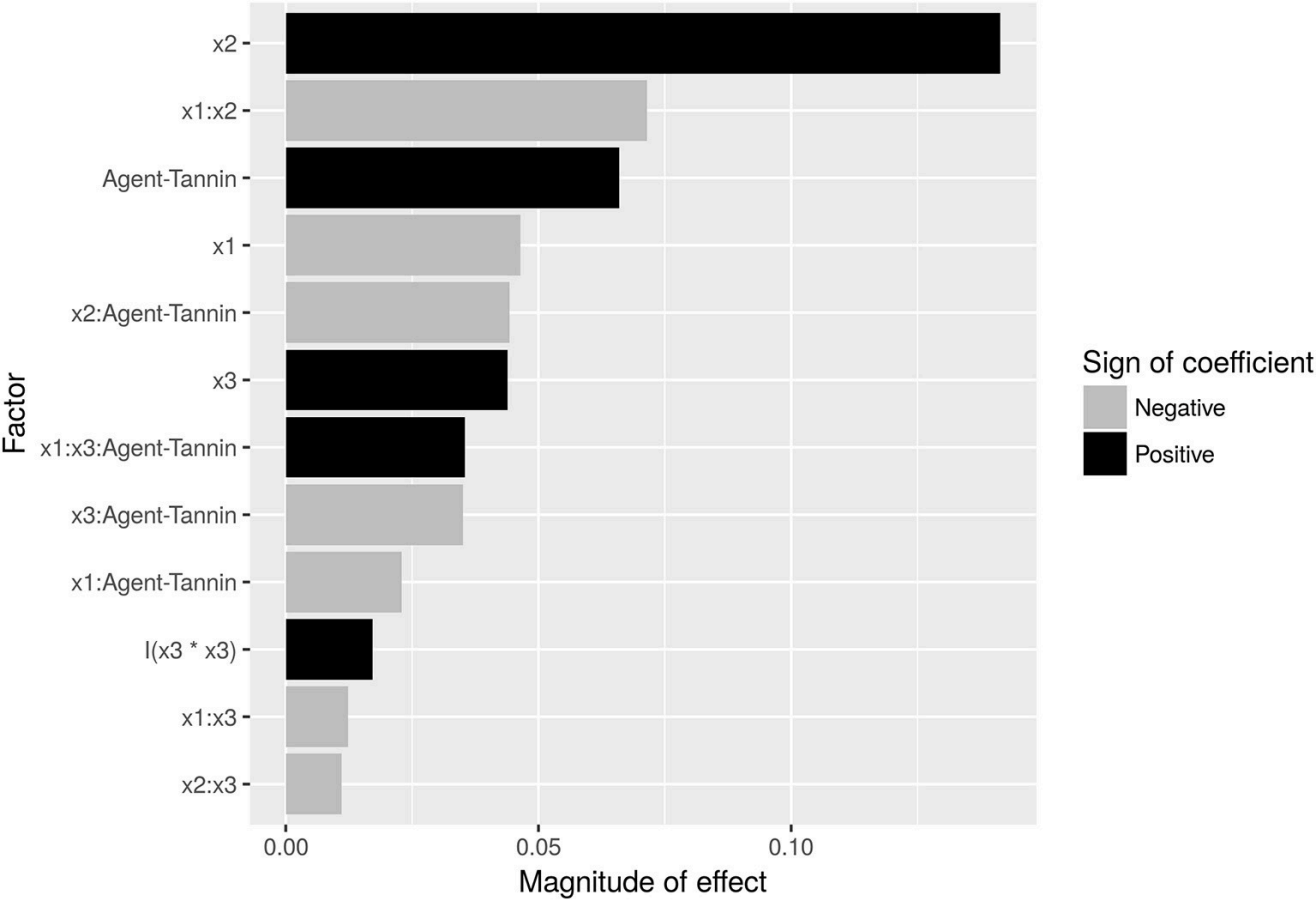
Pareto-plot of effect sizes of factors contributing to the analysis of covariance (ANCOVA) model. Interactions are separated by colons between the respective factors ×1 (pH: 8, 9), ×2 (Concentration: 40, 80 mg/L), ×3 (Time: 120, 145, 170, 195, 220 h), and agent (Flocculation Agent: Chitosan, Tannin). Detailed regression results are shown in Supplemental 8.

FE is dominated by the concentration of employed agent (Figure 4), with an increase from 40 to 80 mg/L flocking agent raising FE by ~14% for both flocculants. However, this can be somewhat offset by a negative interaction effect between flocculant concentration (x2) and pH (x1): FE decreases by 7%, if pH is raised from 8 to 9 while the concentration is raised by the respective amount. This indicates, that an increase in pH (which itself has a negative effect of 4%) cannot be offset by a doubling of flocculant concentration. Overall, modified tannin yields 7% higher FE compared to chitosan (Figure 3A). However, it interacts negatively with concentration. Time (x3) has a minor small positive impact both in a linear and quadratic mode (Figure 4). Interactions are also shown in Figure 3: Time impacts differently on FE depending on the flocking agent, whereas for interactions between agent and flocculant concentration or pH (Figures 3A,B) differences are not significant (α = 0.05).

Flock sizes appeared to be larger for biological flocculants than for pH shifting, which is in line with literature (Vandamme et al., 2012a), as base flocculation causes formation of small precipitates limiting the size of flocs (Supplemental 10).

It has been recognized, that microalgae are associated with species and environment dependent bacterial consortia, which may boost or reduce microalgae biomass yields depending on culture conditions (Fuentes et al., 2016). In addition to the microalgae themselves, algae associated bacteria may secrete flocculation enhancers, such as exopolysaccharides (Nwodo et al., 2012), which in turn could add to flocculation performance.

**Figure 5:**
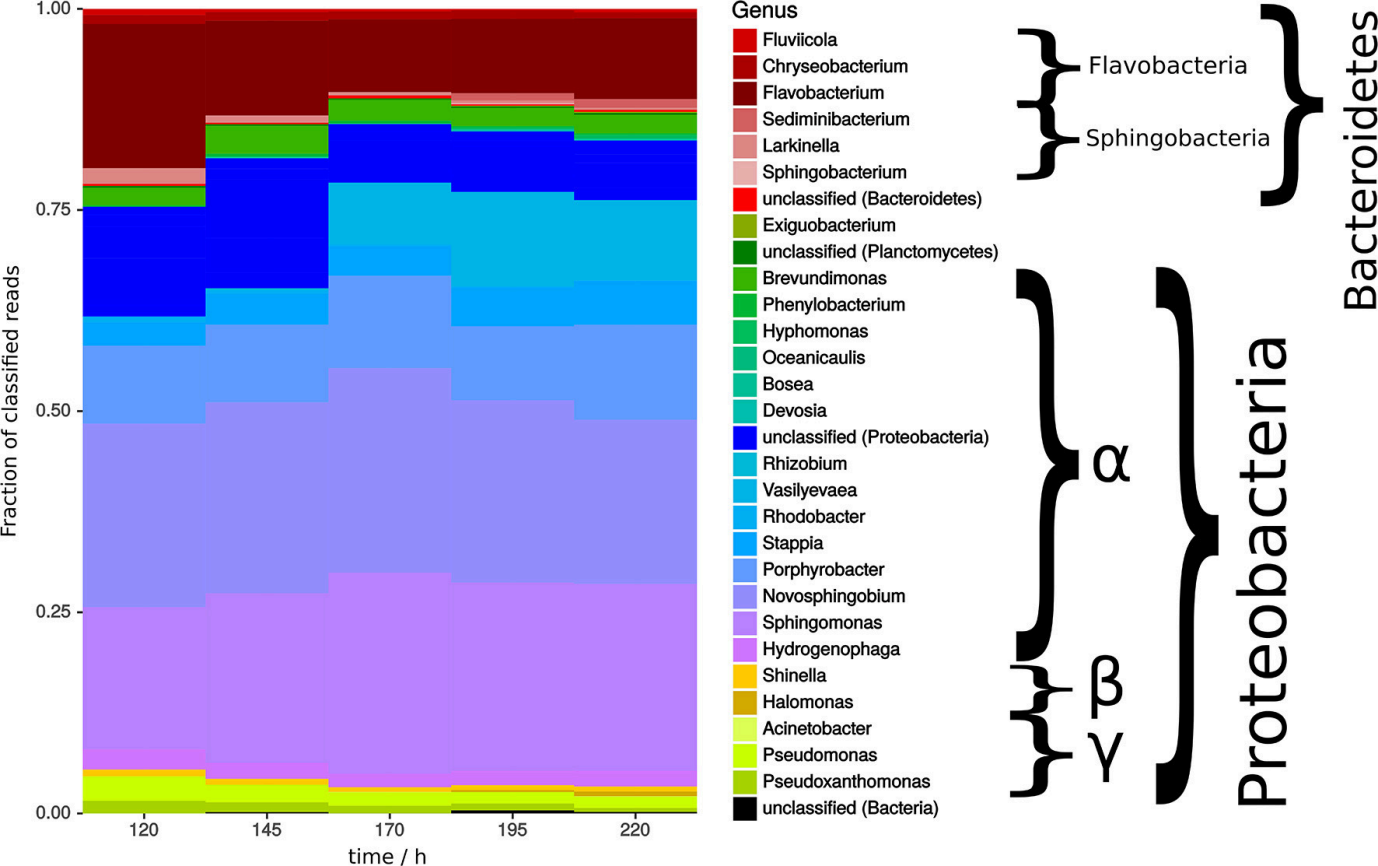
Bacterial community analysis of open pond cultivation over time showing fraction of classified reads. Shades of color indicate class of respective organism with red being Flavobacteria and Sphingobacteria, green and blue being α-proteobacteria, dark yellow being β-proteobacteria, and bright yellow being γ-proteobacteria.

Bacterial communities were mainly dominated by α-proteobacteria and strains of bacteroidetes, in which the former tended to increase and the latter decreased over time (Figure 5). These clades were previously shown to be more likely associated with green algae (Ramanan et al., 2016). However, the overall changes in bacterial communities during the time of cultivation were minor. Notable were the genera Sphingomonas and Novosphingobium, which are described as possible mutualist in the algae cultivation (Borde et al., 2003) and beneficial plant endophyte, respectively (Rangjaroen et al., 2017). Both strains degrade polysaccharides and aromatic compounds. Further, isolates of the genus *Flavobacterium* sp. has previously been described to positively affect the flocculation process (Lee et al., 2013). More specifically, in the absence of bacteria, flocs are still formed but do not sediment anymore (Ramanan et al., 2016).

In summary, time did not have a significant effect during the second half of cultivation time (Figure 5). It could be speculated that absence of time effects might at least in part relate to stability of bacterial communities as bacteria have been shown to play a key role in algae flocculation. Coincidentally, zeta potential of microalgae samples (Supplemental 8) were also constant over time, however it is unknown how the surface potential is possibly shielded by associated microbial communities.

### Effect of Algae Organic Matter

To evaluate the effect of AOM, a set of experiments varying the type of biological flocculant, flocculant concentration, and pH both with and without the presence of AOM was conducted.

**Figure 6:**
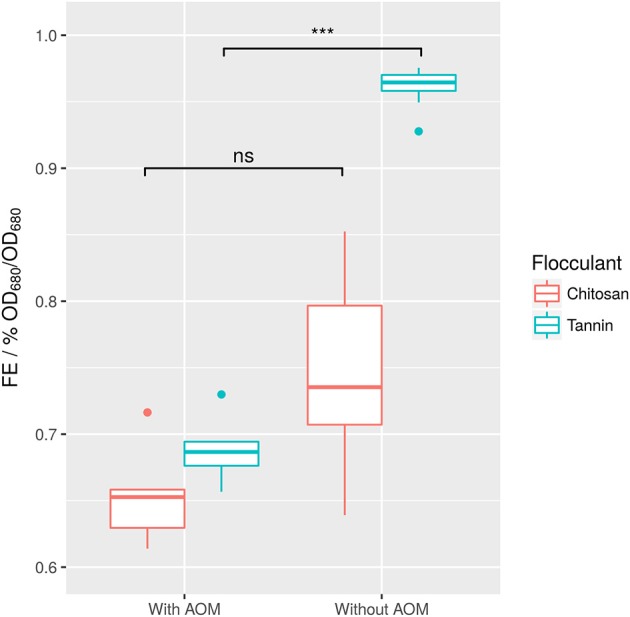
Raw values of tannin- and chitosan-induced FE in dependence of AOM. Difference between samples with and without AOM are not significant for chitosan (difference in means 7% FE), whereas for tannin they are (***p ≤ 0.001, difference in means 28% FE). Each boxplot contains *n* = 8 tests, in which two pH-values, 2 flocculant concentrations, and 2 time points after induction of flocculation were sampled.

Surprisingly, FE mostly depended on the interaction between the agent and the presence of AOM. More accurately, separation of AOM from the sample generally led to a higher FE. However, this increase was much higher for tannin than for chitosan (Figure 6). The strong effect of AOM combined with the independence of FE from time indicates (Supplemental 9), that most of the organic matter affecting flocculation might be produced before the first measurement.

It was previously shown that even low concentrations of AOM may result in a strong increase in required flocculant (Bernhardt et al., 1991). Especially for chitosan, this effect has been described. It has been proposed that negatively charged carboxyl groups on the surface of AOM-polysaccharides interact with soluble metal cations in the medium, which are required for the flocculation process (Vandamme et al., 2012a). However, sterical interactions of polysaccharides with the algae surface may also play a role. In summary, the tested tannin appears to be superior to chitosan in absence of AOM.

### Comparison of Flocculants

AOM has been shown to contain extracellular polysaccharides with negatively charged carboxyl groups, which in turn facilitate interaction with positively charged metal ions in solution (Bernhardt et al., 1991). These ions, especially double-positively charged magnesium, have been shown to be required for efficient flocculation. Dynamic light scattering allowed for comparison of hydrodynamic size as well as zeta potential: Tannin showed both a lower zeta potential (−52.7 ± 2.2 mV) and a larger z-average of 8,016 ± 3,687 nm than chitosan (−34.8 ± 1.4 mV and 106 ± 55 nm). This is possibly derived from the higher number of hydroxyl groups per molecule for the latter. In contrast, larger molecule size might allow for stronger bridging effects and avidity independently of the pH. Considering the improved effectiveness of the tannin-based product in presence of AOM (which captures metal ions required for flocculation), it could be speculated that tannin-based CFL-PT is less dependent on metal ions in its function as flocculant. The presented differences likely account for the discrepancy in induced FE between the two flocking agents.

## Conclusion

Algae harvest from saltwater using flocculation is especially challenging due to its ionic strength. Some flocculants react to this environment by coiling, making flocculation inefficient. In contrast, pH changes require high amounts of added base due to buffering effects of media components. This issue was addressed by comparing different biopolymers for potential use as flocculant. The results of this work emphasize, that any data regarding flocculation efficiencies and derived reported cost are only relevant under given pH, bacterial communities, salt content, algae strain, growth phase, cultivation mode, and AOM content.

**Figure 7:**
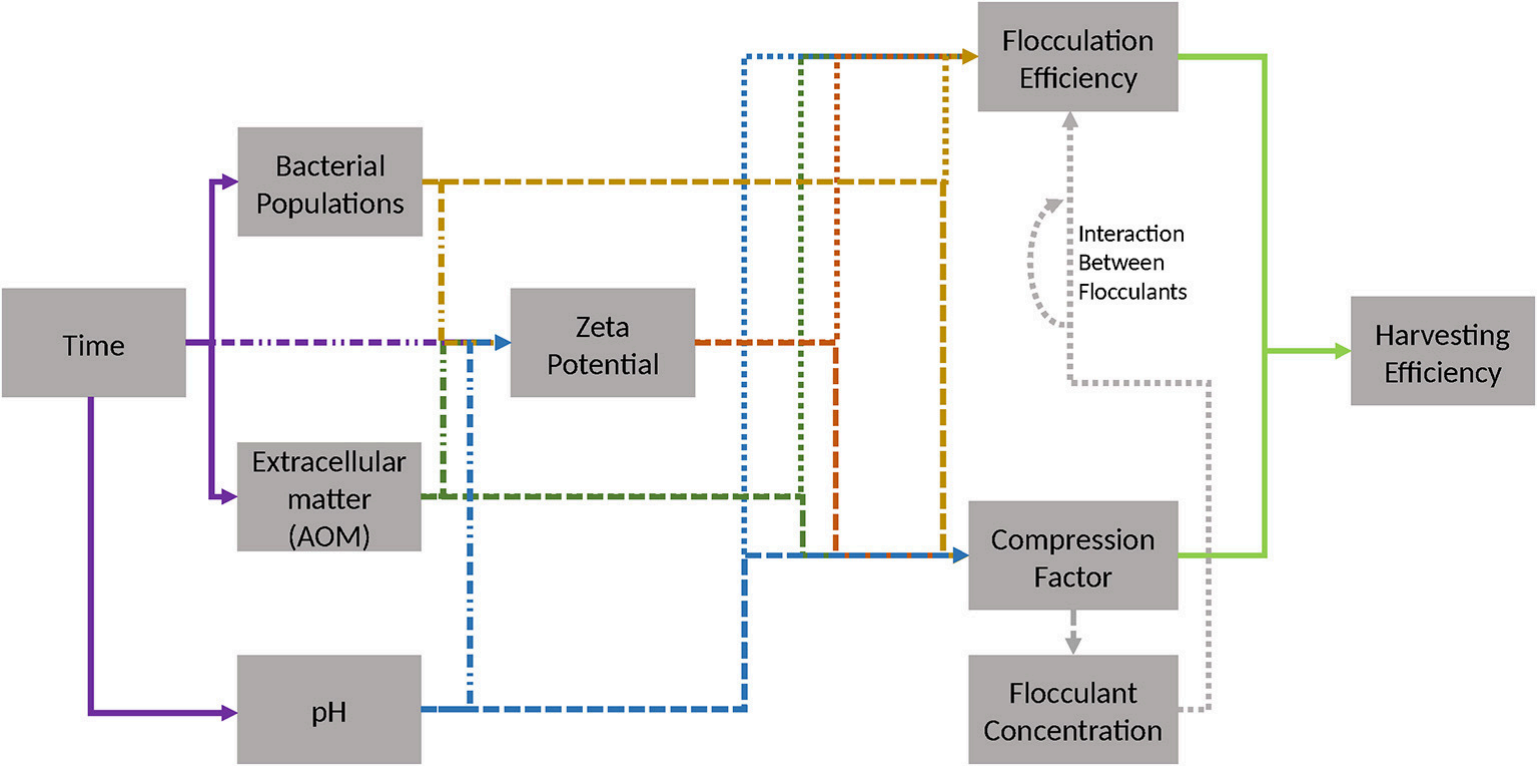
Possible relevant dependencies for evaluation of flocculation. Colors and shapes indicate source and effector. Source of effect: violet—time, yellow—bacterial populations, green—extracellular AOM, blue—pH, gray—flocculant concentration. Effector: dot and dash line—zeta potential, dotted line—FE, dashed line—compression factor. Light green indicates mathematical relationship of FE and compression factor to harvesting efficiency, which is given in section Materials and Methods.

So far, the interdependencies of the algae flocculation procedure (Figure 7) have been only poorly characterized. Thus, this work represents a first step toward a more systematic approach in unveiling the relevant factors. Especially, confounded interaction effects present a challenge in the highly sensitive process step of algae flocculation. Simple multiple linear modeling can be hampered by the inherent censoring of the FE unit. In summary, questions relating to key parameters that influence flocculation for algal biomass harvesting should be posed in a manner, which considers the underlying complex network of interactions.

## Availability of Data and Materials

The datasets generated and/or analyzed during the current study are available from the corresponding author on **r**easonable request.

## Author Contributions

FBr conceived the study design. FBr, NM, IA, TB, and FBo analyzed the data. Flocculation experiments were conducted by FBr, DH, and NF while sequencing and metagenomic analyses were done by DW, AW, and JK. The manuscript was prepared by FBr, IA, NM, and TB.

### Conflict of Interest Statement

TB and FB were employed by company BBSI GmbH. The remaining authors declare that the research was conducted in the absence of any commercial or financial relationships that could be construed as a potential conflict of interest.

## Abbreviations

ANOVA: Analysis of Variance
AOM: Algae Organic Matter
DBC: Dry Biomass Concentration
OD: Optical Density
FE: η, Flocculation Efficiency.
