# Supplementary material for: Harvest of the Oleaginous Microalgae Scenedesmus obtusiusculus by Flocculation From Culture Based on Natural Water Sources
Source: Front Bioeng Biotechnol. 2018 Dec 18;6:200. doi: 10.3389/fbioe.2018.00200 (PMC6305336; doi:10.3389/fbioe.2018.00200)
Supplement: Supplementary file 1 [file Data_Sheet_1.docx]

**Supplemental**

Supplemental 1: Composition of artificial salt water base. Elemental analysis was conducted by ICP using a 700 Series ICP-OES by Agilent Technologies. SO_4_ was determined gravimetrically by barium sulfate precipitation. Remaining mass was assigned to Cl.

| Component | Proportion /  mg/g artificial salt water |
| --- | --- |
| Na | 340 |
| Mg | 32 |
| Ca | 10.2 |
| K | 8.6 |
| B | 0.94 |
| Cl | 540 |
| SO_4_ | 68 |

Supplemental 2: OD_680_ / Biomass correlation for *S. obtusiusculus*. Samples were washed twice before determination of dry biomass. The corresponding regression formula is

DBM / g/l = 0.0028+0.418 * OD_680_ with an R^2^ of 0.994.

Supplemental 2: Raw values of autoflocculation induced by supplementation of different bases. The number on top of each plot displays the sedimentation time in hours after induction of flocculation.


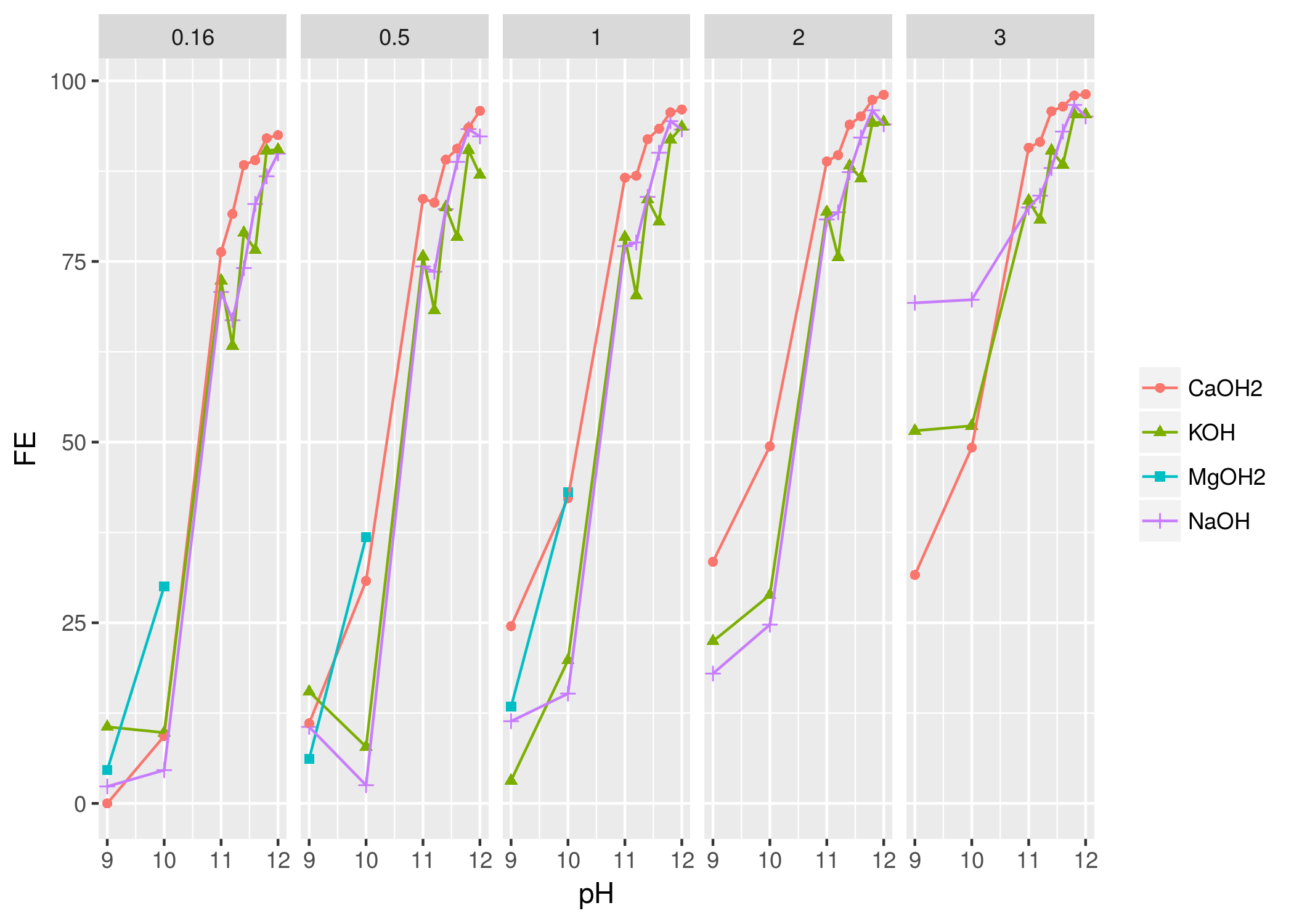


Supplemental 3: Parameters of multiple linear regressions with different bases in dependence of time and pH according to the formula FE=t+ph

| Coefficients:  Estimate Std. Error t value Pr(>\|t\|)  (Intercept) -0.384287 0.201044 -1.911 0.097549 .  t 0.029726 0.008387 3.544 0.009414 **  ph 0.108819 0.017439 6.240 0.000428 ***  Residual standard error: 0.02757 on 7 degrees of freedom  Multiple R-squared: 0.8803, Adjusted R-squared: 0.8462  F-statistic: 25.75 on 2 and 7 DF, p-value: 0.0005926 | **Ca(OH)_2_** |
| --- | --- |
| Coefficients:  Estimate Std. Error t value Pr(>\|t\|)  (Intercept) -0.774683 0.149559 -5.180 0.00128**  t 0.030084 0.006239 4.822 0.00192**  ph 0.138003 0.012973 10.638 1.42e-05***  Residual standard error: 0.02051 on 7 degrees of freedom  Multiple R-squared: 0.9512, Adjusted R-squared: 0.9372  F-statistic: 68.21 on 2 and 7 DF, p-value: 2.569e-05 | **KOH** |
| Coefficients:  Estimate Std. Error t value Pr(>\|t\|)  (Intercept) -1.00432 0.14024 -7.162 0.000183 ***  t 0.02714 0.00585 4.639 0.002372 **  ph 0.15810 0.01216 12.997 3.71e-06 ***  Signif. codes: 0 ‘***’ 0.001 ‘**’ 0.01 ‘*’ 0.05 ‘.’ 0.1 ‘ ’ 1  Residual standard error: 0.01923 on 7 degrees of freedom  Multiple R-squared: 0.9645, Adjusted R-squared: 0.9544  F-statistic: 95.23 on 2 and 7 DF, p-value: 8.389e-06 | **NaOH**  Signif. codes:  0 ‘***’  0.001 ‘**’  0.01 ‘*’  0.05 ‘.’  0.1 ‘ ’  1 |

Supplemental 4: Raw values of autoflocculation induced by supplementation of chitosan to cells cells grown in ABV and BG11 medium. The number on top shows pH, whereas the number on the right hand side shows the employed concetration of flocculant in mg/L.


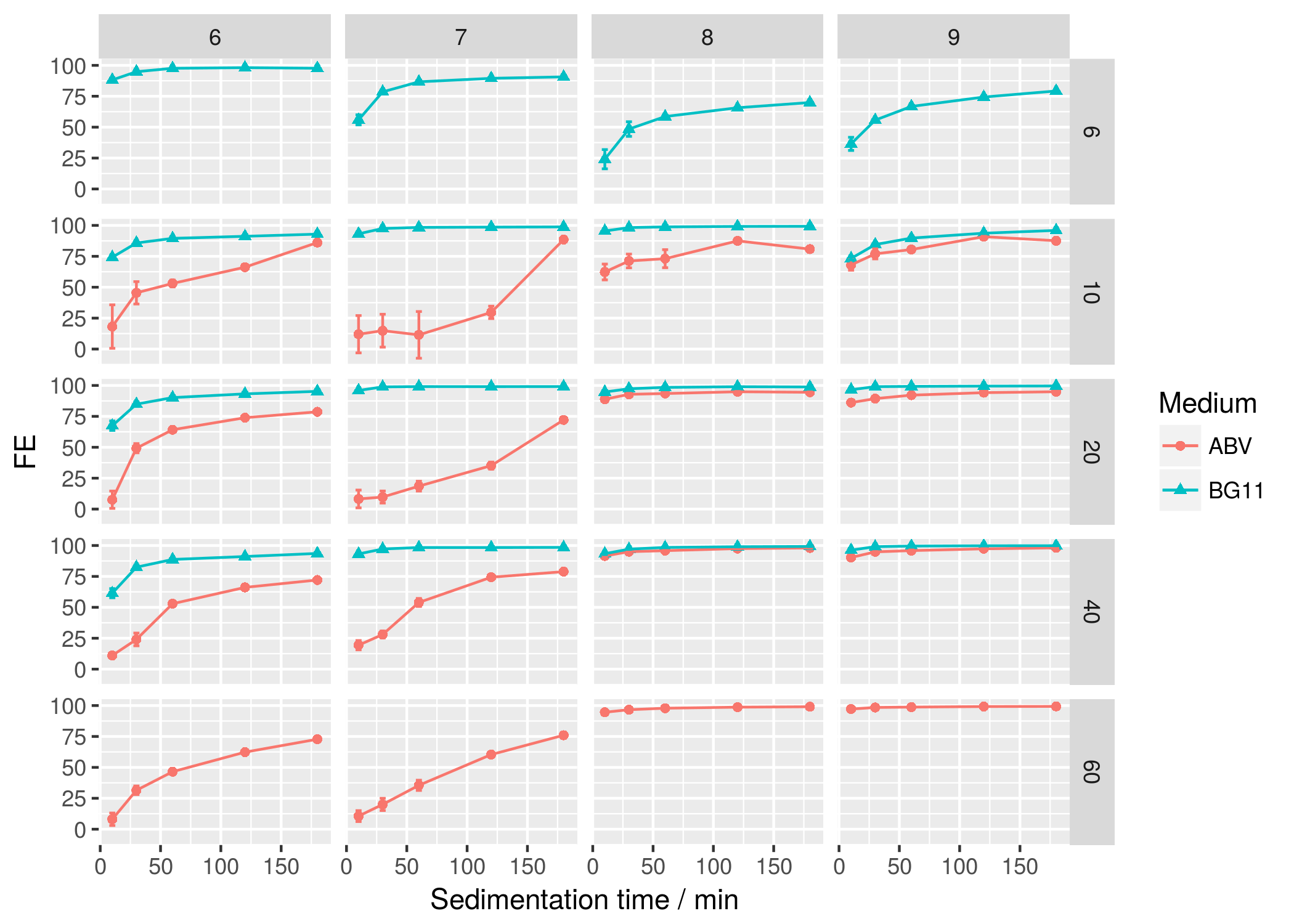


Supplemental 5: Raw values of autoflocculation induced by supplementation of chitosan and tannin to cells grown in ABV medium. The number on top shows pH, whereas the number on the right hand side shows the employed concetration of flocculant in mg/L.


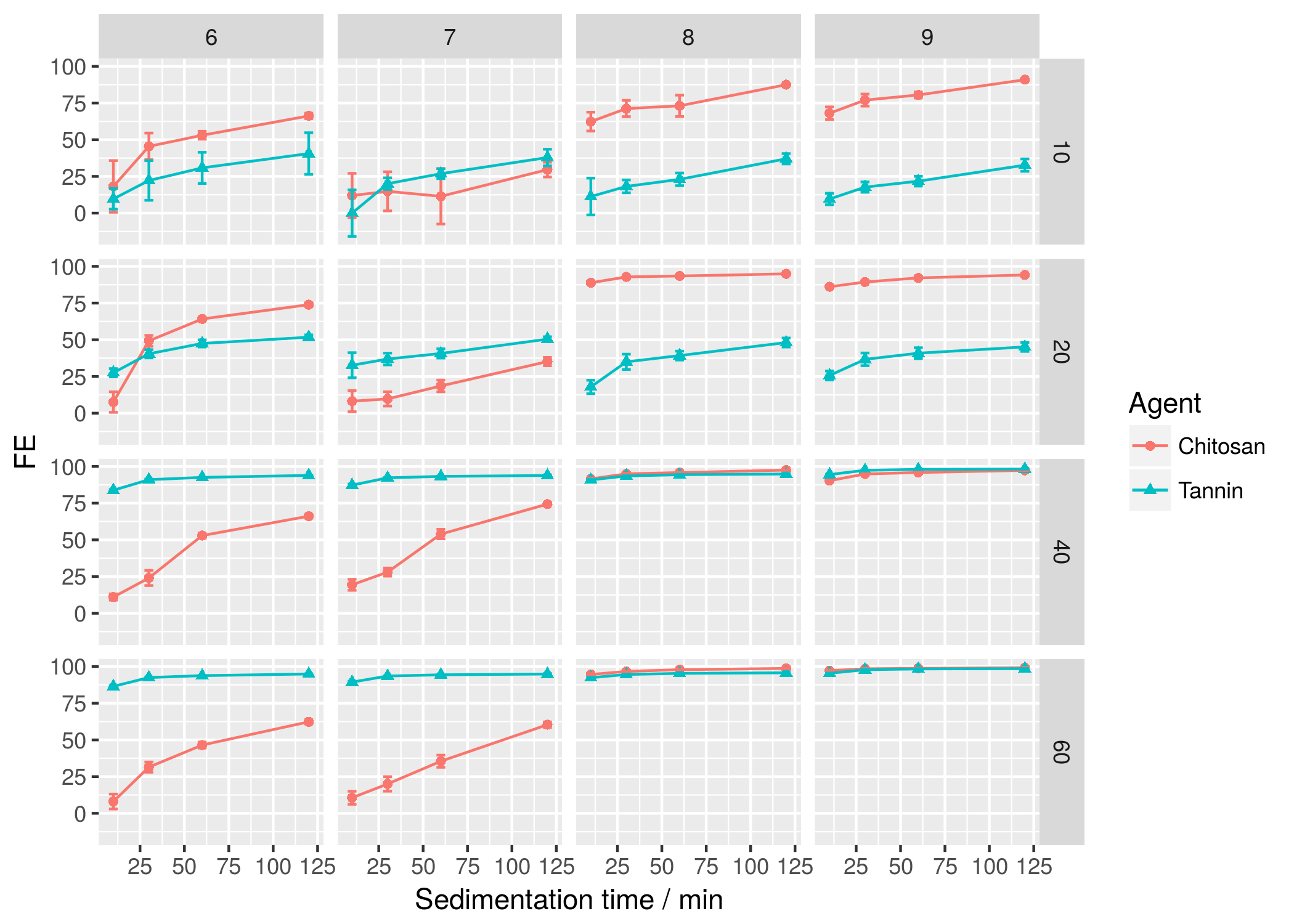


Supplemental 6: Growth of *Scenedesmus obtusiusculus* A189 in artificial sea salt medium. Estimated parameters as shown by Gompertz fit (blue) are μ:0.0628/h λ:32.3981 h and A:10.82623. Dashed red lines show time points of flocculation experiments.

**
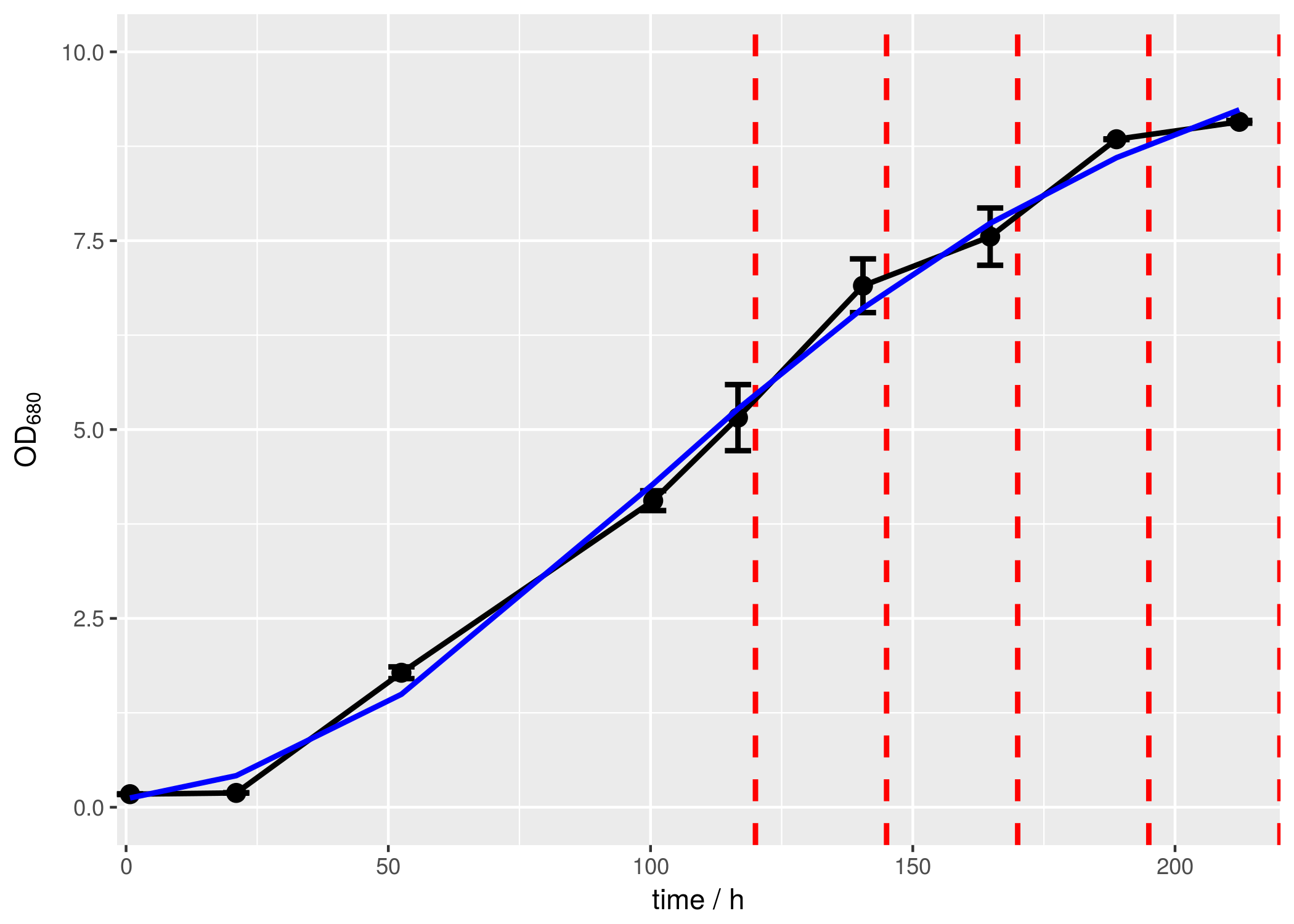
**

Supplemental 7: Top: AIC reduced ANCOVA model parameters with factors x1 (pH: 8, 9), x2 (Concentration: 40, 80 mg/l), x3 (Time: 120, 145, 170, 195, 220 h) and agent (Flocculation Agent: Chitosan, Tannin). Bottom: Regression analytics for the respective model. Normality of the dataset was not rejected (Shapiro-Wilk, α=0.05).

| FE ~ x1 + x2 + x3 + Agent + I(x3 * x3) + x1:x2 + x1:x3 + x2:x3 + x1:Agent + x2:Agent + x3:Agent + x1:x3:Agent |
| --- |
| Coefficients: Estimate Std. Error t value Pr(>\|t\|)  (Intercept) 0.506247 0.016893 29.969 < 2e-16 ***  x1 -0.046499 0.020156 -2.307 0.02896 *  x2 0.141410 0.020156 7.016 1.53e-07 ***  x3 0.043886 0.009200 4.770 5.65e-05 ***  Agent-Tannin 0.066001 0.020156 3.274 0.00290 **  I(x3 * x3) 0.017231 0.003477 4.955 3.43e-05 ***  x1:x2 -0.071518 0.023274 -3.073 0.00480 **  x1:x3 -0.012327 0.011637 -1.059 0.29887  x2:x3 -0.011023 0.008229 -1.340 0.19154  x1:Agent-Tannin -0.022985 0.023274 -0.988 0.33213  x2:Agent-Tannin -0.044306 0.023274 -1.904 0.06767 .  x3:Agent-Tannin -0.035021 0.011637 -3.009 0.00562 **  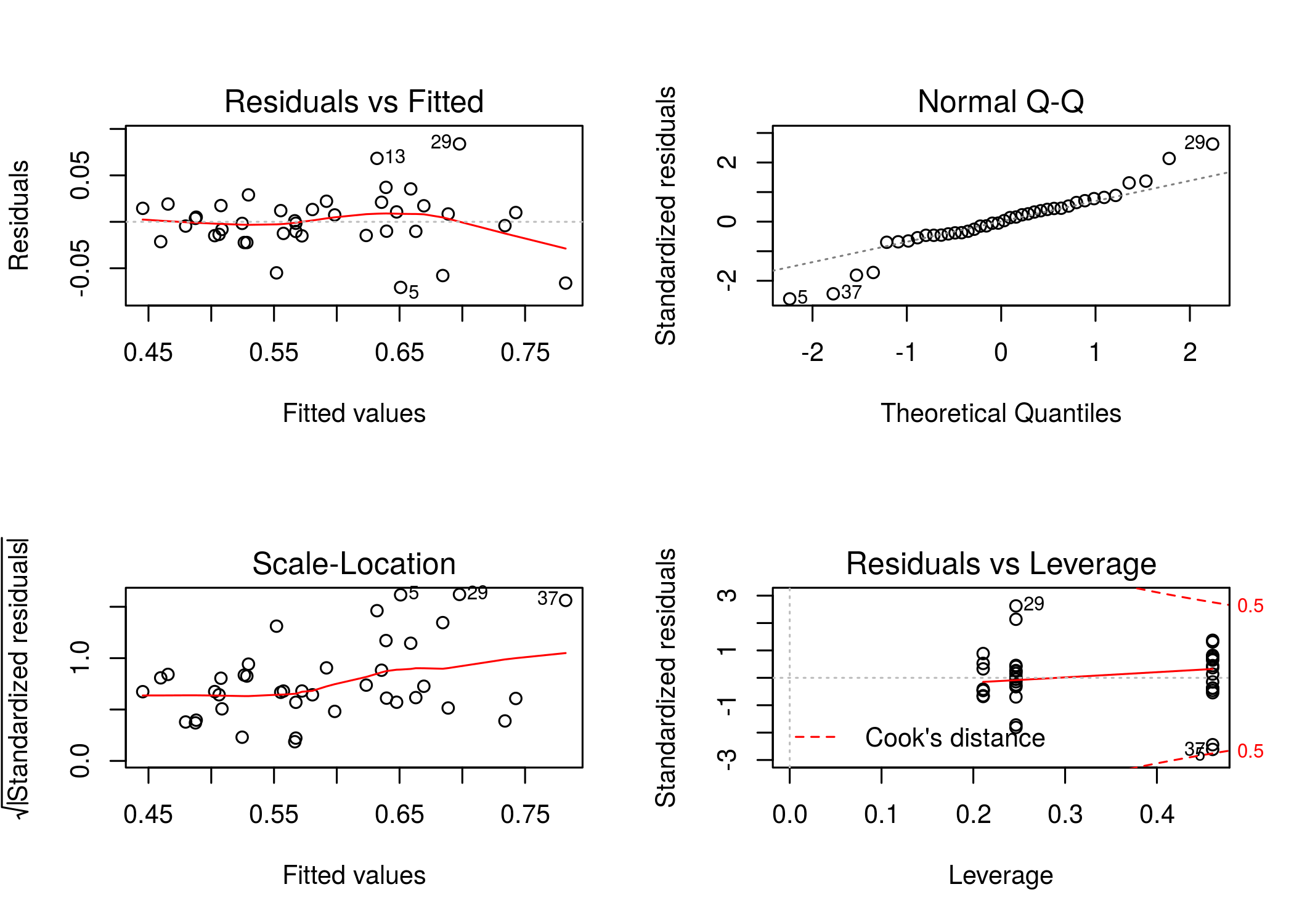x1:x3:Agent-Tannin 0.035447 0.016457 2.154 0.04034 * |
| Significance levels: 0 ‘***’ 0.001 ‘**’ 0.01 ‘*’ 0.05 ‘.’ 0.1 ‘ ’ 1 |
| Residual standard error: 0.0368 on 27 degrees of freedom  Multiple R-squared: 0.8845, Adjusted R-squared: 0.8332  F-statistic: 17.23 on 12 and 27 DF, p-value: 1.253e-09 |

Supplemental 8: Zeta potential of culture over time.


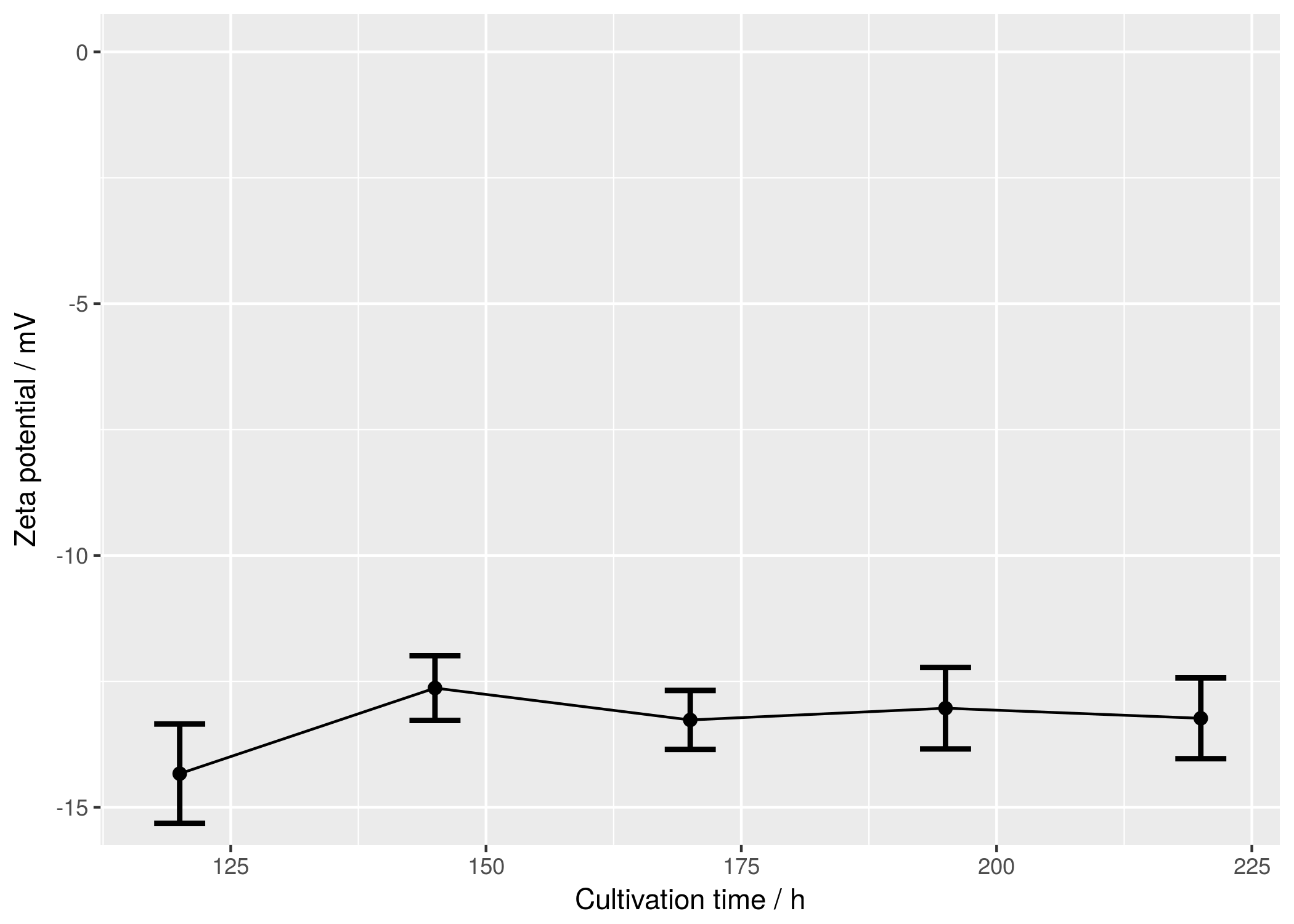


Supplemental 9: ANCOVA Parameters… XXXX

| lm(formula=FE ~ x1 + x2 + x3 + FM + AOM + x1:x2 + FM:AOM, data=FlokN7aom) |
| --- |
| Coefficients:  Estimate Std. Error t value Pr(>\|t\|)  (Intercept) 0.582097 0.036234 16.065 2.42e-14 ***  x1 -0.099971 0.036234 -2.759 0.01092 *  x2 0.002651 0.036234 0.073 0.94228  x3 0.043659 0.012811 3.408 0.00231 **  FMTannin 0.029286 0.036234 0.808 0.42689  AOMWO 0.092081 0.036234 2.541 0.01792 *  x1:x2 0.071914 0.051243 1.403 0.17331  FMTannin:AOMWO 0.244319 0.051243 4.768 7.49e-05 *** |
| Signif. codes: 0 ‘***’ 0.001 ‘**’ 0.01 ‘*’ 0.05 ‘.’ 0.1 ‘ ’ 1 |
| Residual standard error: 0.07247 on 24 degrees of freedom  Multiple R-squared: 0.8618, Adjusted R-squared: 0.8215  F-statistic: 21.38 on 7 and 24 DF, p-value: 7.247e-09 |

Supplemental 10: Comparison of flocs emerging from different flocculation agents using bright field microscopy at 1000x magnification (NaOH (A), Chitosan (B), Tannin (C) and Control of culture before flocculation (D). Images were taken using a Zeiss Axiolab A1 and scale bars were added using Zeiss Zen software v2.3 SP1.


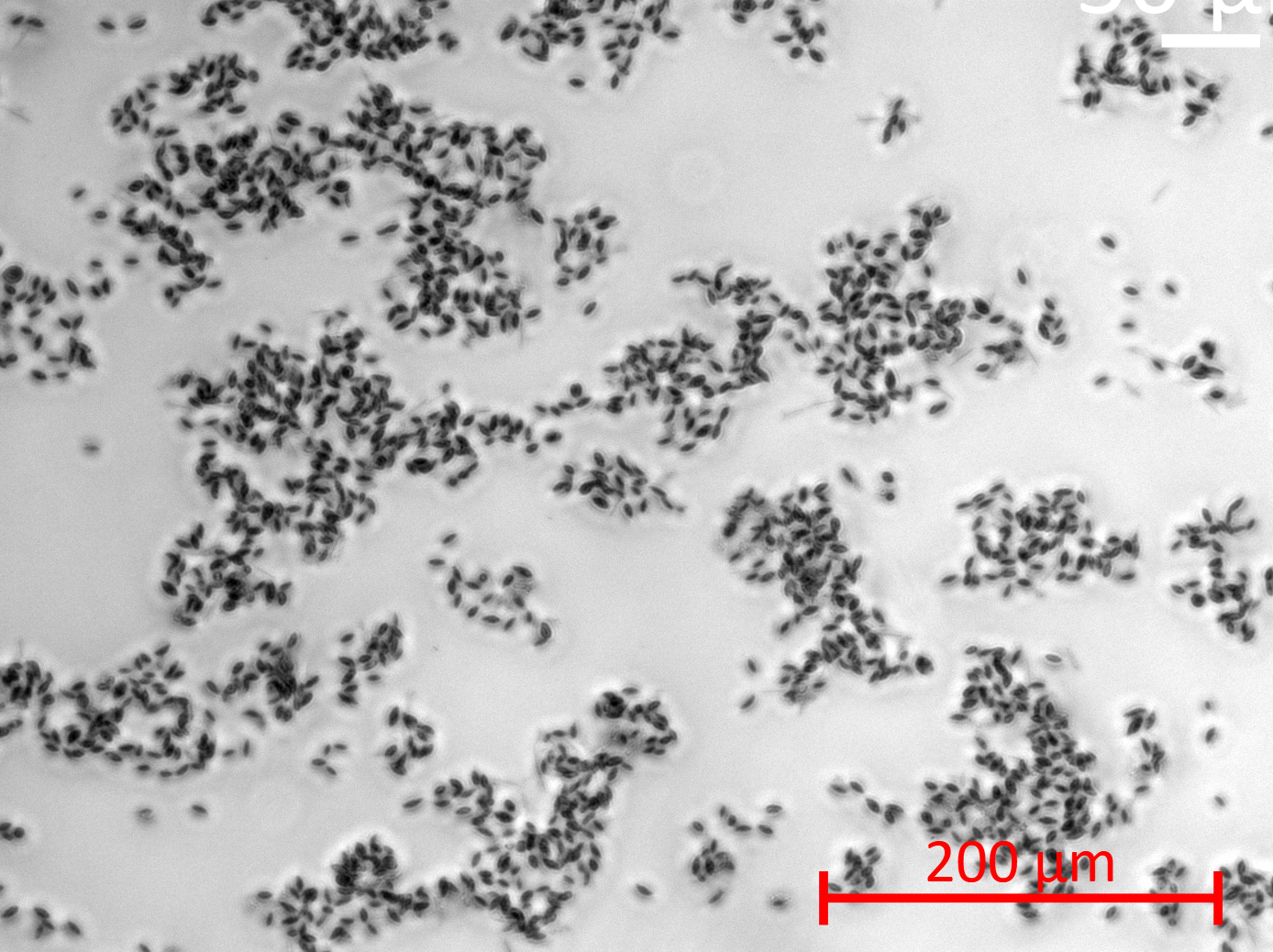

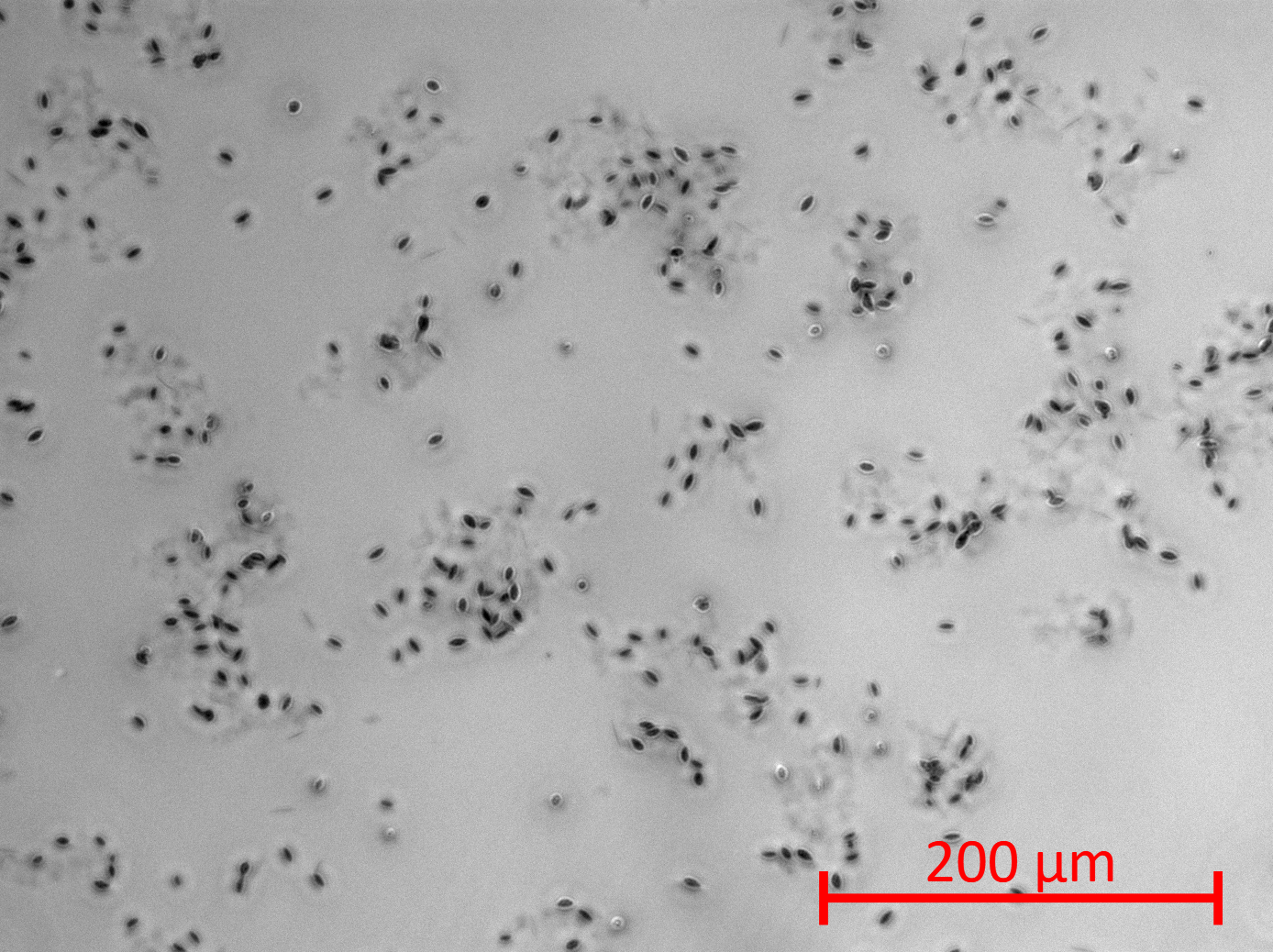


**A B**

**
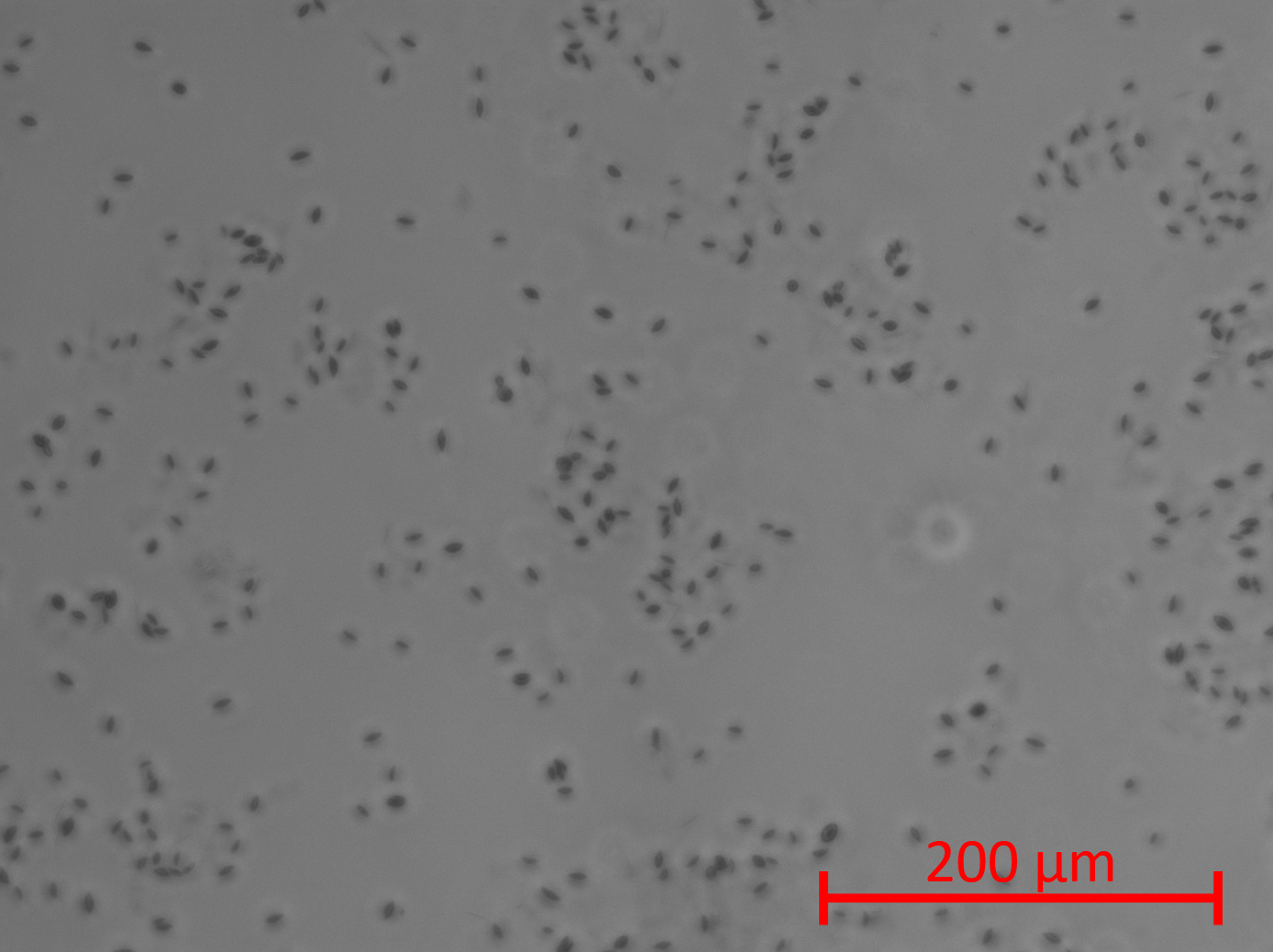

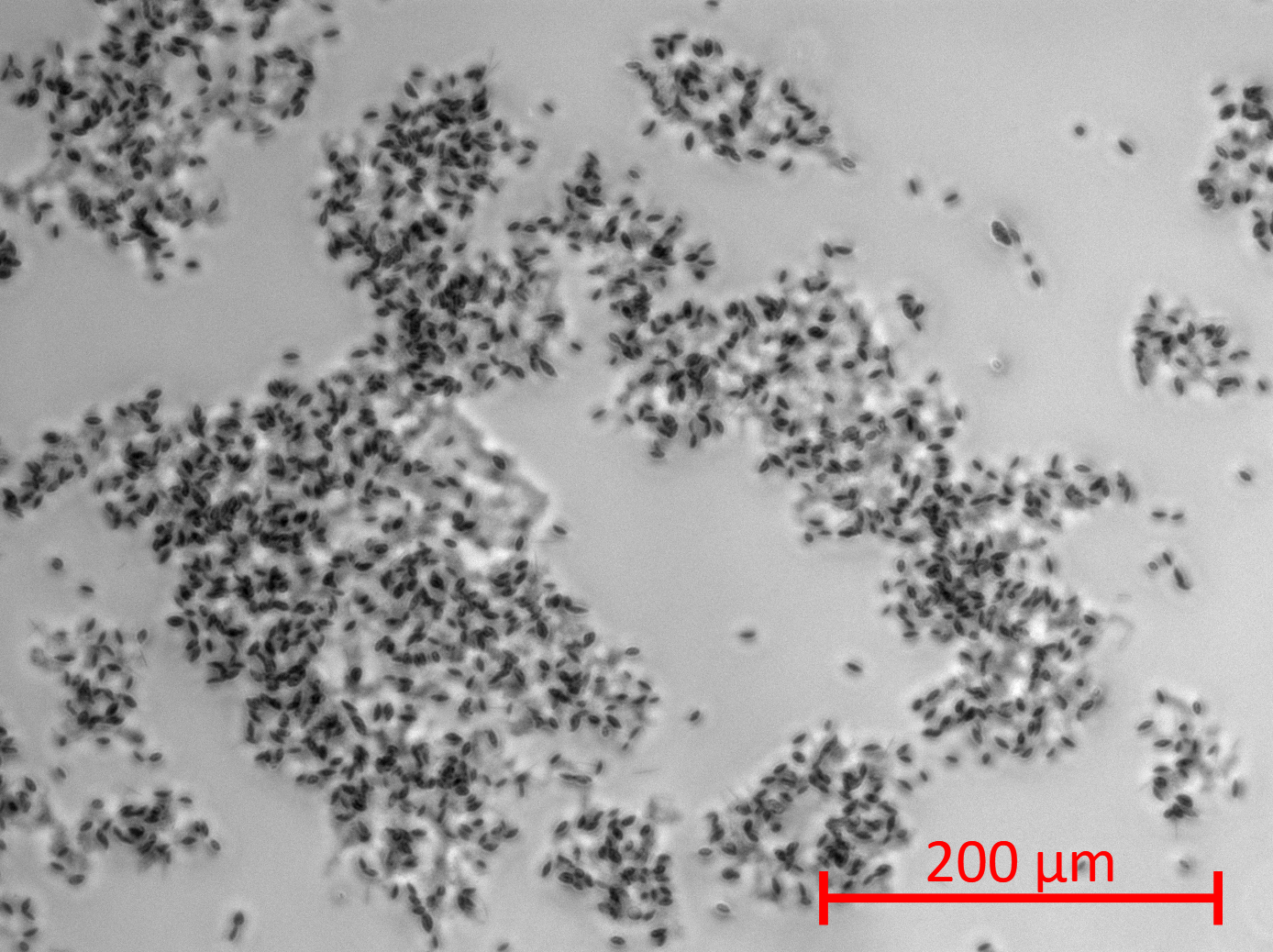
**

**C D**
